# Fingolimod Improves Anxiety-like Behavior and Modulates Sphingosine-1-Phosphate Receptors Gene Expression in a Diabetic Mouse Model

**DOI:** 10.3390/biom15111485

**Published:** 2025-10-22

**Authors:** Przemysław Leonard Wencel, Kamilla Blecharz-Klin, Agnieszka Piechal, Justyna Pyrzanowska, Dagmara Mirowska-Guzel, Robert Piotr Strosznajder

**Affiliations:** 1 Laboratory of Preclinical Research and Environmental Agents, Mossakowski Medical Research Institute, Polish Academy of Sciences, 5 Pawinskiego St., 02-106 Warsaw, Poland; rstrosznajder@imdik.pan.pl; 2 Department of Experimental and Clinical Pharmacology, Centre for Preclinical Research and Technology CePT, Medical University of Warsaw, 1B Banacha St., 02-097 Warsaw, Poland; kamilla.blecharz-klin@wum.edu.pl (K.B.-K.); agnieszka.piechal@wum.edu.pl (A.P.); jpyrzanowska@wum.edu.pl (J.P.); dagmara.mirowska-guzel@wum.edu.pl (D.M.-G.)

**Keywords:** diabetes, sphingosine-1-phosphate, sphingosine kinases, fingolimod, FTY720, inflammation, cytokines, brain cortex, hippocampus, behavior

## Abstract

Background: Type 2 diabetes mellitus (T2DM) is a rapidly expanding worldwide health issue associated with impairments in memory and executive functions. The bioactive sphingolipid sphingosine-1-phosphate (S1P) regulates cell death/survival and the inflammatory response by acting on S1P receptors (S1PRs). Unfortunately, the role of S1PRs signaling in the T2DM brain remains elusive. Methods: The effect of fingolimod (FTY720, S1PRs modulator) on the behavior and expression profile of genes encoding S1PRs, sphingosine kinases (SPHK1 and 2), glucose transporters, proteins engaged in insulin signaling, sirtuin 1 (SIRT1), and proinflammatory cytokines in the brain cortex and hippocampus of diabetic mice was examined. Results: We observed a significant reduction in *S1pr1*, *Sirt1*, and insulin-like growth factor-1 (*Igf1*) gene expression that was accompanied by elevation of *Sphk2*, *S1pr3*, *Il6*, and *Tnf* in T2DM mice. Moreover, animals showed anxiety-like behavior and memory deficits. Fingolimod administration recovered downregulated *S1pr1*, *Sirt1*, and *Igf1* expression and upregulated *Slc2a4* (GLUT-4) and *Ide* (insulin-degrading enzyme). Furthermore, FTY720 reduced the elevated expression of *Il6* and *Tnf*. Fingolimod also exerted an anxiolytic effect in T2DM. Conclusions: Results indicate an important role of S1PR modulation in T2DM. Moreover, fingolimod affected mRNA levels of proteins engaged in glucose metabolism/insulin signaling and improved the behavior of diabetic mice.

## 1. Introduction

Type 2 diabetes mellitus (T2DM) is a metabolic disorder that affects millions worldwide and is a pressing concern for the global public health system. In 2024, about 589 million adults were estimated to be living with diabetes, and 90% of them were accounted for by T2DM [1]. Insulin resistance and alterations in glucose and lipid (including free fatty acids (FFAs), cholesterol, and sphingolipids) metabolism have been implicated in the pathogenesis of T2DM [2]. Emerging data suggest that changes in the concentration of circulating bioactive sphingolipids are characteristic of diabetes, and some sphingolipids can act as predictors of the development of T2DM [3,4,5]. In T2DM, bioactive sphingolipids, such as ceramide, are usually connected with pathological changes in pancreatic β-cells and insulin signaling pathways, whereas a derivative of sphingosine, sphingosine-1-phosphate (S1P), has been associated with either pro- or anti-apoptotic effects depending on the stimulatory conditions and type of tissue [6]. The S1P signaling axis has emerged as a critical regulator of neuronal survival, neuroinflammation, and synaptic function, thereby influencing emotional and cognitive behaviors [7]. It exerts its effects through five specific G-protein-coupled receptors (S1PR1–S1PR5) [8]. Accumulating data indicate that bioactive sphingolipid signaling may be involved in the pathology of both type 1 and T2DM, as well as in the development of micro- and macro-vascular complications, behavioral changes, and memory impairment [6,9,10].

Anxiety and mood disorders are frequently observed comorbidities in patients with diabetes mellitus, exacerbating the disease burden and complicating glycemic control. Experimental diabetic models, particularly in rodents, consistently show increased anxiety-like behaviors, which are often associated with neurochemical imbalances, neuroinflammation, and oxidative stress within the central nervous system (CNS) [11,12,13]. Fingolimod (FTY720), a functional modulator of S1P receptors, approved for multiple sclerosis (MS) treatment, crosses the blood–brain barrier and exerts neuroprotective and anti-inflammatory effects that may mitigate behavioral abnormalities, including anxiety and depression-like phenotypes [14,15,16,17]. The findings reported by Gammoh and coworkers (2023) suggest that fingolimod therapy may be associated with a reduction in depressive symptoms among patients with multiple sclerosis experiencing elevated stress levels [18]. These results highlight the potential mental health benefits of fingolimod in this patient population and underscore the importance of further research into the CNS effects of disease-modifying therapies, particularly in the context of psychological stress. Despite its therapeutic potential, the specific effects of fingolimod on anxiety-like behavior and its modulation of S1P receptor gene expression in the context of diabetes-induced neuropathology remain underexplored.

In this study, we investigated the effects of fingolimod on behavioral outcomes and gene expression in the cerebral cortex and hippocampus of a mouse model of T2DM. Specifically, we analyzed the expression of genes encoding S1P receptors, sphingosine kinases, glucose transporters, insulin signaling-related proteins, the neuroprotective sirtuin 1, and proinflammatory cytokines. Dysregulation of the S1P/S1PR signaling axis has been implicated in various neurodegenerative and inflammatory disorders; however, its specific role in the diabetic brain remains largely unexplored. Understanding these mechanisms may open novel approaches for treating neuropsychiatric complications of diabetes.

## 2. Materials and Methods

### 2.1. Animal Model and Treatment

All procedures were approved by the II Local Ethics Committee for Animal Experimentation in Warsaw (approval No. WAW2/065/2019) and conducted in compliance with the EU Directive 2010/63/EU and following the ARRIVE guidelines. Male C57BL/6J mice (aged 10–12 weeks, weighing 27 ± 2 g) purchased from The Animal House of the Mossakowski Medical Research Institute PAS in Warsaw, Poland, were fed a high-fat diet (HFD; Ssniff Spezialdiäten GmbH, Soest, Germany: 60 kJ% fat, 20 kJ% protein, 20 kJ% carbohydrates, E15742-34, equivalent to Research Diets, Inc., Göttingen, Germany, D12492) for 16 weeks. The control group received a standard chow diet (SD; Ssniff: 9 kJ% fat, 24 kJ% protein, 67 kJ% carbohydrates). Mice were individually housed in plastic cages within an air-conditioned environment featuring mechanical ventilation, a controlled 12 h light–dark cycle, temperature maintained between 20 and 24 °C, and humidity at 55% (±10%). Environmental enrichment, including cotton rolls and wooden fibers for nest building, was provided in all cages. At the study onset, there were no significant differences in body weight between the groups. The T2DM model was created by i.p. injection of HFD mice with 75 mg/kg streptozotocin (Sigma-Aldrich, St Louis, MO, USA) after 8 weeks on the high-fat diet, followed three days later by a second dose of streptozotocin (50 mg/kg b.w.). SD mice received appropriate control (0.1 M citrate buffer, pH = 4.5). Mouse blood glucose levels were measured after 12 weeks of the experiment following an overnight fast of 14–16 h, and animals with blood glucose ≥ 11.1 mM (200 mg/dL) were considered diabetic (STZ). After 96 days, the animals began receiving daily intraperitoneal injections of FTY720 (1 mg/kg b.w., Cayman Chemical Company, Ann Arbor, MI, USA) dissolved in 0.9% NaCl or vehicle (NaCl solution) once daily for 2 weeks.

One day following the final FTY720 injection, the animals were euthanized by decapitation. The brain cortex and hippocampus were quickly isolated on an ice-cold glass Petri dish and stored at −80 °C for qPCR analysis. The numbers of animals used for the gene expression study and behavioral studies are presented in Table 1.

**Figure 1:**
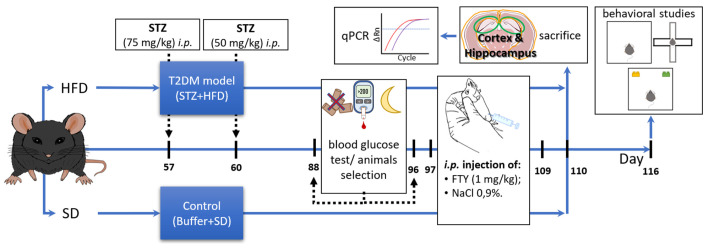
Schematic diagram representing the timeline for experiments using the diabetic mouse model.

Behavioral assessments commenced four days after the last FTY720 dose, beginning with an open field test, and were conducted daily from 9:00 to 16:00 over three consecutive days (see Figure 1).

### 2.2. Intraperitoneal Glucose Tolerance Test

Animals were fasted overnight for 16 h (6:00 p.m.–10:00 a.m.) before the glucose tolerance test. Blood glucose levels were measured from the tail vein with an Accu-Check Performa glucometer (Roche Diabetes Care GmbH, Mannheim, Germany) immediately prior to glucose (2 g/kg b.w./i.p., dissolved in saline) injection (0 min) and at 15, 30, 60, and 120 min after glucose administration.

### 2.3. Gene Expression Analysis

RNA was extracted from tissues using TRI-reagent, following the manufacturer’s instructions, and DNA was digested with DNase I (Sigma-Aldrich/Merck, St Louis, MO, USA). Then, 4 μg of total RNA was reverse-transcribed using a High Capacity Reverse Transcription Kit (Thermo Fisher Scientific Baltics UAB, Vilnius, Lithuania). Real-time PCR was performed on Applied Biosystems 7500 Real-Time PCR System (Thermo Fisher Scientific, Inc., Waltham, MA, USA) using TaqMan Fast Advanced Master Mix, following the manufacturer’s guidelines and using specific mouse primers (Applied Biosystems, Life Technologies Corporation life technologies corporation, Pleasanton, CA, USA): *Sphk1* (Mm00448841_g1), *Sphk2* (Mm00445021_m1) *S1pr1* (Mm02619656_s1), *S1pr3* (Mm02620181_s1), *Il6* (Mm00446190_m1), *Tnf* (Mm00443258_m1), *Ide* (Mm00473077_m1), *Igf1* (Mm00439560_m1), *Insr* (Mm01211875_m1), *Slc2a3* (Mm00441483_m1), *Slc2a4* (Mm00436615_m1), *Sirt1* (Mm01168521_m1). Each sample was analyzed in triplicate to quadruplicate. Gene expression was calculated using the ΔΔCt method and normalized against beta-actin (*Actb*, Mm00607939_s1), and the results were expressed as relative quantities (RQs).

### 2.4. Behavioral Tests

The behavioral experiments were conducted in a blinded manner and adhered to the ARRIVE guidelines (https://arriveguidelines.org accessed on 20 September 2020). Observers who were unaware of the experimental groups and conditions scored the animals’ behavior. The animals’ activities were recorded using a video camera positioned above the testing apparatus, and the footage was analyzed with Noldus EthoVision XT10 software (ver.10.1.856). Data analysis was performed by researchers who were not involved in designing the study. Each behavioral test was followed by a 24 h rest period. To ensure proper acclimation, mice were transferred from the housing room to the experimental room at least one hour before testing each day. Additionally, the mice were well-handled before the behavioral assessments, with daily handling sessions lasting 1–2 min conducted for up to two weeks prior to testing.

#### 2.4.1. Open Field (OF) Test

The open field (OF) test was first introduced by Hall (1934) as a method to evaluate locomotor activity and exploratory behavior in unfamiliar surroundings [19]. For this study, naive animals were used in the OF test to minimize habituation effects and avoid any carryover from repeated exposure to the apparatus. Each mouse was placed individually in the center of a grey open arena measuring 57 cm by 57 cm, enclosed by 50 cm high walls with an open top. The OF setup was situated in a soundproof experimental room with diffused lighting. The test lasted 8 min, during which behaviors such as climbing, grooming, total distance traveled, velocity, and time spent in the center area of the arena were recorded. To maintain the sensitivity of the test, no observers were present in the room during testing. After the session, the mouse was removed and the apparatus was cleaned with a 10% ethanol solution to eliminate any odor cues. The same arena was also used the following day for the novel object recognition test.

#### 2.4.2. Novel Object Recognition (NOR) Test

The novel object recognition (NOR) test was originally developed for rats by Ennaceur and Delacour in 1988 and has since been successfully adapted for use with mice [20,21,22,23]. This test is widely used as a relatively low-stress behavioral assay to examine various aspects of learning and memory in rodents. It is an effective method for evaluating different memory phases, such as acquisition, consolidation, and recall [24]. NOR specifically assesses episodic memory and recognition memory deficits, which affect the ability to distinguish between novel and familiar objects [25,26], and is useful for detecting neuropsychological changes [24]. Additionally, the NOR test can investigate spatial object memory (object location memory) [27]. During the task, memory consolidation occurs, involving the processing of spatial and contextual features of objects in different brain regions [28].

The test was conducted using the previously described open field (OF) apparatus and consisted of three stages: habituation, familiarization, and choice. Following optimized experimental protocols, the habituation phase took place during the OF test, where each animal freely explored the empty arena for 8 min. On the second day, during the familiarization phase, animals were placed facing the wall opposite two identical objects (A1 and A2, Lego towers) positioned in opposite corners of the arena and allowed to explore freely for 8 min. Afterward, the mice were removed, and both the apparatus and objects were cleaned with a 10% ethanol solution to remove scent cues.

Two hours later, in the choice phase, one of the familiar objects was replaced with a novel object (object B, a glass bottle), differing in color, shape, and structure. Each mouse was placed near the center of the wall opposite the objects and given 8 min to explore. Exploration was defined as the mouse’s head coming within 2 cm of an object. The animals’ behavior was recorded by a camera positioned above the apparatus and analyzed using specialized software.

Several parameters were measured: time spent exploring each object during familiarization (t_A1_, t_A2_) and choice phases (t_A1_, t_B_), total exploration time during familiarization (t_A1A2_) and choice (t_A1B_), and overall exploration time [t_total_ = (t_A1A2_+ t_A1B_)]. Discrimination indices were calculated to assess preference during familiarization [DI_A1A2_ = (t_A1_ − t_A2_)/(t_A1_ + t_A2_)] and during choice [DI_A1B_ = (t_B_ − t_A1_)/(t_B_ + t_A1_)]. The global habituation index (GHI), which compares total exploration time between familiarization and choice phases, was also determined [GHI = t_A1A2_/t_A1B_]. Lastly, the recognition index (RI), representing the proportion of time spent exploring the novel object relative to total exploration, was calculated [RI = t_B_/t_total_].

$${DI}_{A1A2}=\frac{t_{A1}-t_{A2}}{t_{A1}+t_{A2}},\;\;\;\;\;\;{DI}_{A1B}=\frac{t_{B}-t_{A1}}{t_{B}+t_{A1}},\;\;\;\;\;\;GHI=\frac{t_{A1A2}}{t_{A1B}},\;\;\;\;\;\;RI=\frac{t_{B}}{t_{total}}$$

#### 2.4.3. Elevated Plus Maze (EPM) Test

On the third day, after the OF and NOR tests were completed, an anxiety and exploration assessment was performed using the elevated plus maze (EPM). This test evaluates anxiety-related behaviors by observing rodents’ instinctive exploratory actions in new settings and their natural avoidance of open, elevated areas [29]. The EPM apparatus consisted of four narrow gray arms (31 cm long, 6.5 cm wide, and 16 cm high), elevated approximately 50 cm above the floor. Two opposite arms were enclosed by gray walls (closed arms) and connected through a central platform to two open arms without walls. The test took place in a soundproof room with diffused lighting. Each mouse was positioned at the center intersection of the arms, facing an open arm, and allowed to explore for 8 min. After the session, the animal was removed, and the maze was cleaned using a 10% ethanol solution to remove any scent traces.

### 2.5. Statistical Analysis

The mRNA expression levels (RQ), blood glucose levels, animal weight, and behavioral results are presented as the means ± SEMs. Data, depending on experimental design, were analyzed using Student’s *t* test or analysis of variance (one-way ANOVA) followed by a Tukey’s post hoc test for multiple comparisons. Effect sizes (η^2^ for ANOVA) were also calculated. Values of η^2^ = 0.14, 0.06, and 0.01 were considered for large, medium, and small effects, respectively. The statistical analyses were performed using GraphPad Prism 6 (GraphPad Software, San Diego, CA, USA). Statistical significance was accepted at *p* < 0.05.

## 3. Results

### 3.1. Animal Weight and Blood Glucose Levels

**Figure 2:**
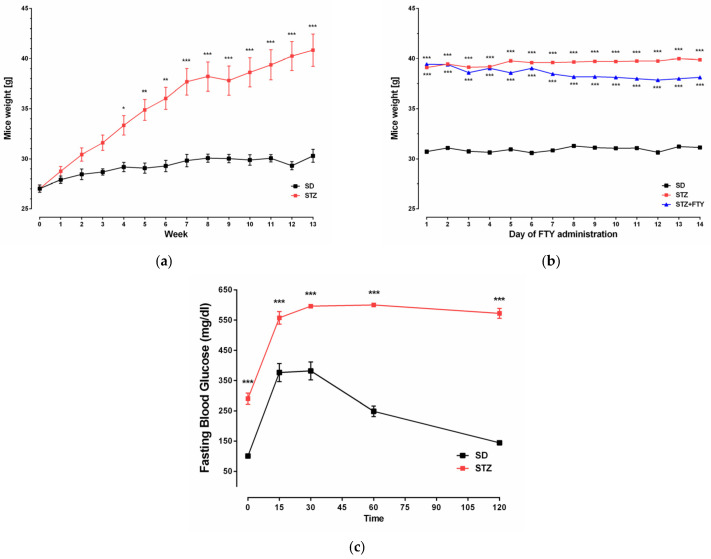
Effect of HFD and STZ on animal body weight [g] and blood glucose levels. Animal weight from beginning of the experiment until glucose test ((**a**) SD: *n* = 8, HFD: *n* = 16) and during administration of FTY720 ((**b**), *n* = 8 per group); IPGTT after 16 h fast ((**c**), *n* = 8 per group). For statistical comparison, Student’s *t* test or one-way ANOVA with Tukey post hoc test was used. *p* values < 0.05 were considered statistically significant (* *p* < 0.05; ** *p* < 0.01; *** *p* < 0.001 as compared to the SD mice).

In diabetic mice generated in our model, we observed a significant increase in body mass. This effect was accompanied by elevated blood glucose levels in STZ mice (Figure 2).

### 3.2. Gene Expression

#### 3.2.1. Gene Expression of Sphingosine Kinases and S1P Receptors in the Brain Cortex and Hippocampus of Diabetic Mice

**Figure 3:**
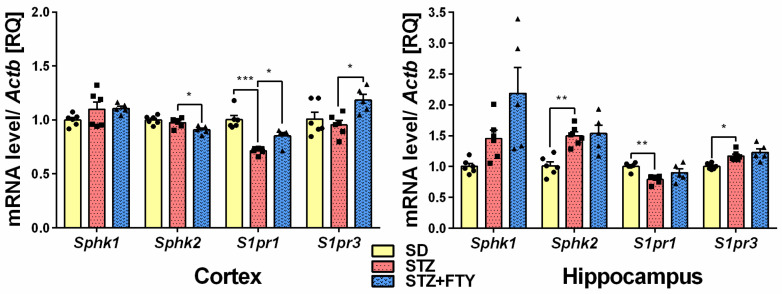
Gene expression levels of sphingosine kinases (*Sphk1* and *2*) and sphingosine-1-phosphate receptors (*S1pr1* and *3*) in the brain cortex and hippocampus of animals on standard diet (SD), animals receiving only high-fat diet and low doses of streptozotocin (STZ) and STZ mice treated with FTY720 (STZ + FTY). * *p* < 0.05; ** *p* < 0.01; *** *p* < 0.001 as compared to the appropriate controls (*n* = 17 for each gene, 5–6 for each group); one-way ANOVA with Tukey post hoc test.

In the hippocampus of diabetic mouse brains, we observed significant upregulation of sphingosine kinase 2 (*Sphk2* (SD: 1.010 ± 0.063, STZ: 1.498 ± 0.066, STZ + FTY: 1.538 ± 0.133, F(2,14) = 11.59, *p* = 0.001, η^2^ = 0.624)) and sphingosine-1-phosphate receptor 3 (*S1pr3* (SD: 1.001 ± 0.020, STZ: 1.173 ± 0.032, STZ + FTY: 1.229 ± 0.059, F(2,14) = 10.03, *p* = 0.002, η^2^ = 0.589)) gene expression with concomitant reduction in sphingosine-1-phosphate receptor 1 (*S1pr1*) levels in both the brain cortex (SD: 1.003 ± 0.037, STZ: 0.715 ± 0.012, STZ + FTY: 0.851 ± 0.035, F(2, 14) = 24.94, *p* < 0.0001, η^2^ = 0.781) and hippocampus (SD: 1.002 ± 0.026, STZ: 0.791 ± 0.026, STZ + FTY: 0.900 ± 0.063, F(2, 14) = 7.705, *p* = 0.006, η^2^ = 0.524). The expression of *Sphk1* was slightly upregulated in both brain structures; however, this upregulation was insignificant. Fingolimod successfully reversed downregulation of *S1pr1* in the brain cortex. The administration of FTY720 reduced the gene expression of *Sphk2* and evoked upregulation of *S1pr3* gene in the brain cortex (*Sphk2* (SD: 1.001 ± 0.016, STZ: 0.974 ± 0.017, STZ + FTY: 0.909 ± 0.017, F(2,14) = 7.541, *p* = 0.006, η^2^ = 0.519); *S1pr3* (SD: 1.010 ± 0.063, STZ: 0.954 ± 0.041, STZ + FTY: 1.184 ± 0.052, F(2,14) = 4.769, *p* = 0.026, η^2^ = 0.405)) (Figure 3).

#### 3.2.2. mRNA Levels of the Proinflammatory Cytokines, Sirtuin 1, Glucose Transporters, and Proteins Engaged in Insulin Signaling in the Brain Cortex and Hippocampus of T2DM Mice

**Figure 4:**
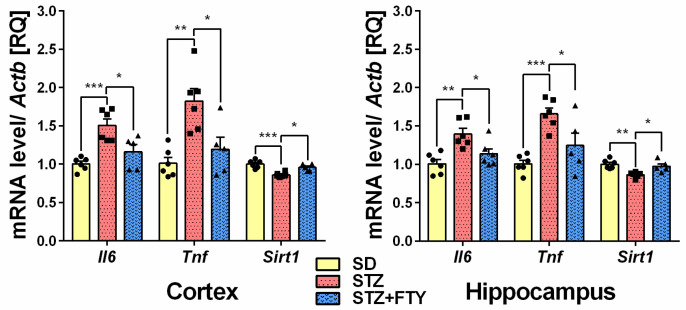
mRNA expression levels of genes encoding proinflammatory cytokines IL-6, TNF and sirtuin 1 (SIRT1) in the brain cortex and hippocampus of standard (SD) mice, streptozotocin (STZ) mice, and STZ mice administered with FTY720 (STZ + FTY). * *p* < 0.05; ** *p* < 0.01; *** *p* < 0.001 as compared to the appropriate controls (*n* = 17 for each gene, 5–6 for each group); one-way ANOVA with Tukey post hoc test.

**Figure 5:**
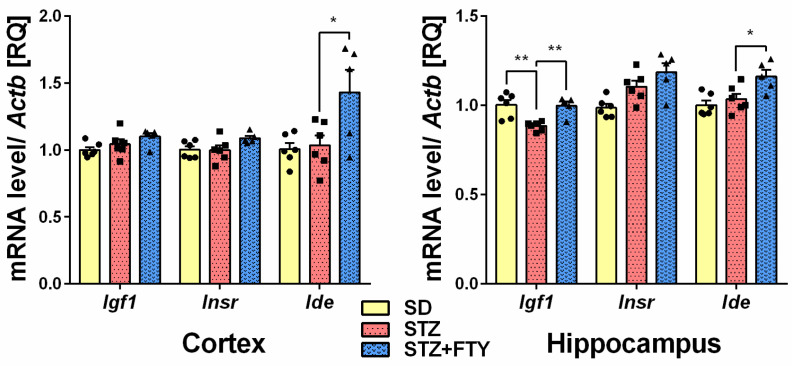
Effect of FTY720 on the mRNA expression levels of proteins engaged in insulin signaling (IGF-1, INSR, and IDE) measured using real-time PCR in the brain cortex and hippocampus of standard (SD) mice, streptozotocin (STZ) mice, and STZ mice administered with FTY720 (STZ + FTY). * *p* < 0.05; ** *p* < 0.01 as compared to the appropriate controls (*n* = 17 for each gene, 5–6 for each group); one-way ANOVA with Tukey post hoc test.

**Figure 6:**
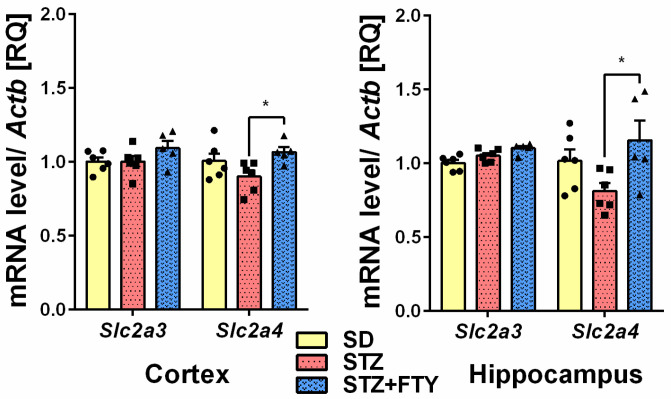
Effect of FTY720 on the glucose transporter (*Slc2a3* and *4*) expression levels measured using real-time PCR in the brain cortex and hippocampus of standard (SD) mice, streptozotocin (STZ) mice, and STZ mice administered with FTY720 (STZ + FTY). * *p* < 0.05 as compared to the appropriate controls (*n* = 17 for each gene, 5–6 for each group); one-way ANOVA with Tukey post hoc test.

Gene expression of proinflammatory cytokines interleukin 6 (*Il6*) and tumor necrosis factor α (*Tnf*) was significantly upregulated in in the brain cortex (*Il6* (SD: 1.003 ± 0.035, STZ: 1.507 ± 0.084, STZ + FTY: 1.160 ± 0.096, F(2,14) = 12.95, *p* = 0.001, η^2^ = 0.649); *Tnf* (SD: 1.014 ± 0.076, STZ: 1.823 ± 0.161, STZ + FTY: 1.195 ± 0.156, F(2,14) = 10.43, *p* = 0.002, η^2^ = 0.599)) and hippocampus (*Il6* (SD: 1.008 ± 0.055, STZ: 1.396 ± 0.072, STZ + FTY: 1.135 ± 0.068, F(2,15) = 9.231, *p* = 0.002, η^2^ = 0.552); *Tnf* (SD: 1.006 ± 0.047, STZ: 1.656 ± 0.077, STZ + FTY: 1.246 ± 0.161, F(2,14) = 11.86, *p* = 0.001, η^2^ = 0.629)) of diabetic mice (Figure 4). Moreover, we observed significant downregulation of sirtuin 1 (*Sirt1*) gene expression in both examined brain structures (cortex (SD: 1.001 ± 0.021, STZ: 0.861 ± 0.013, STZ + FTY: 0.958 ± 0.020, F(2,14) = 16.05, *p* = 0.001, η^2^ = 0.696); hippocampus (SD: 1.002 ± 0.027 STZ: 0.868 ± 0.016, STZ + FTY: 0.980 ± 0.034, F(2,14) = 8.122, *p* = 0.005, η^2^ = 0.537)) that was followed by a reduction in insulin-like growth factor-1 (*Igf1*) gene expression in the hippocampus (SD: 1.002 ± 0.028, STZ: 0.884 ± 0.011, STZ + FTY: 0.998 ± 0.024, F(2,14) = 9.834, *p* = 0.002, η^2^ = 0.584). The administration of FTY720 reversed those changes (Figure 4 and Figure 5). The expression of *Insr* and *Slc2a3* remained unchanged in both examined structures of STZ mice before and after FTY720 treatment. Additionally, fingolimod elevated the expression of *Ide* (insulin-degrading enzyme) and *Slc2a4* (solute carrier family 2 (facilitated glucose transporter), member 4) in both the cortex (*Ide* (SD: 1.005 ± 0.046, STZ: 1.034 ± 0.073, STZ + FTY: 1.431 ± 0.166, F(2,14) = 5.411, *p* = 0.018, η^2^ = 0.436); *Slc2a4* (SD: 1.006 ± 0.050, STZ: 0.900 ± 0.042, STZ + FTY: 1.066 ± 0.033, F(2,14) = 3.762, *p* = 0.049, η^2^ = 0.350)) and hippocampus (*Ide* (SD: 1.002 ± 0.025, STZ: 1.035 ± 0.030, STZ + FTY: 1.162 ± 0.037, F(2,14) = 7.188, *p* = 0.007, η^2^ = 0.507); *Slc2a4* (SD: 1.015 ± 0.077, STZ: 0.811 ± 0.054, STZ + FTY: 1.155 ± 0.133, F(2,14) = 3.749, *p* = 0.050, η^2^ = 0.349)) (Figure 5 and Figure 6).

### 3.3. Behavioral Tests

#### 3.3.1. Open Field (OF)

**Figure 7:**
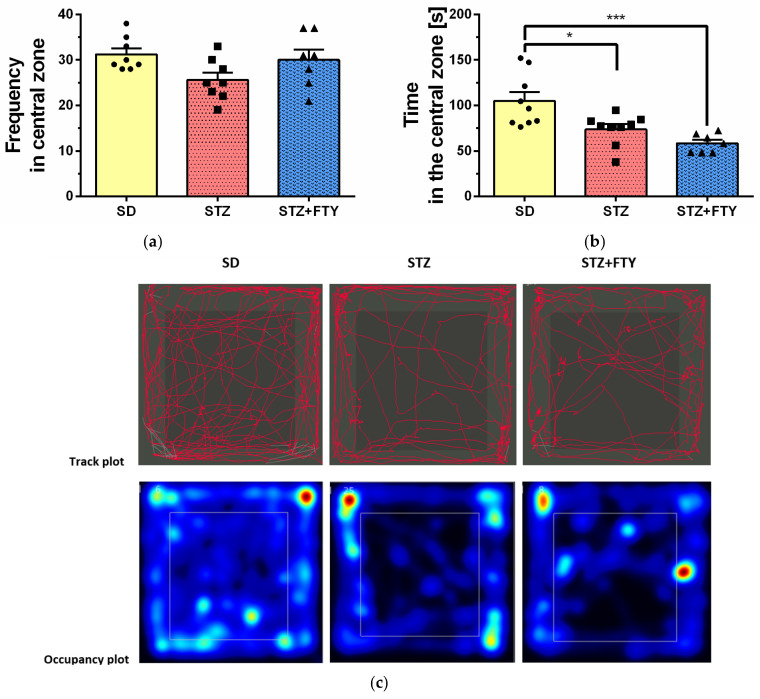
Number of entries into the central zone of the open field (**a**), duration spent in the central area of the testing arena (**b**), and representative movement paths along with heat maps of occupancy (**c**) for mice fed a standard diet (SD, *n* = 9), mice on a high-fat diet treated with low-dose streptozotocin (STZ, *n* = 9), and mice additionally treated with FTY720 (STZ + FTY, *n* = 7). * *p* < 0.05; ; *** *p* < 0.001 compared to respective control groups (one-way ANOVA followed by Tukey’s post hoc test).

**Figure 8:**
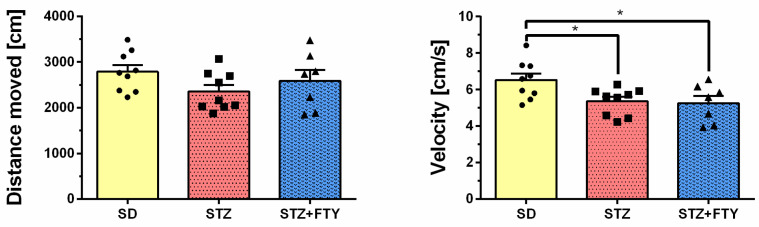
Key locomotor parameters in the open field test for mice fed a standard diet (SD, *n* = 9), those receiving a high-fat diet combined with low-dose streptozotocin treatment (STZ, *n* = 9), and animals additionally treated with FTY720 (STZ + FTY, *n* = 7). * *p* < 0.05 compared to the respective control groups (one-way ANOVA with Tukey’s post hoc test).

**Figure 9:**
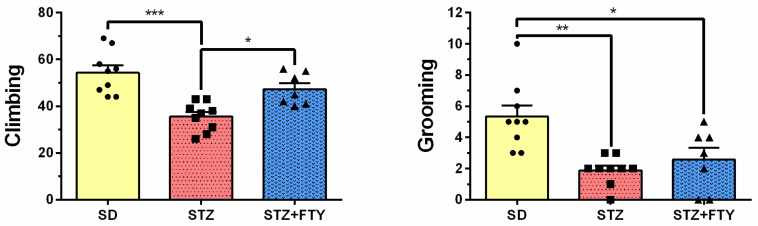
Frequency of exploratory behaviors in the open field test, including number of climbing and grooming episodes, for mice fed a standard diet (SD, *n* = 9), mice on a high-fat diet treated with low-dose streptozotocin (STZ, *n* = 9), and mice additionally treated with FTY720 (STZ + FTY, *n* = 7). * *p* < 0.05; ** *p* < 0.01; *** *p* < 0.001 compared to respective control groups (one-way ANOVA with Tukey’s post hoc test).

The open field (OF) test is a widely used assay that allows for the assessment of exploratory behavior, anxiety-like responses, and locomotor activity in mice. Statistical analysis revealed significant differences between groups in the amount of time spent in the central zone of the arena (SD: 104.8 ± 9.712, STZ: 73.77 ± 5.620, STZ + FTY: 58.44 ± 3.910, F(2,22) = 10.37, *p* = 0.001, η^2^ = 0.485). Mice treated with streptozotocin (STZ) spent significantly less time in the central area compared to animals from the standard diet (SD) control group, with this effect being less pronounced in the group receiving STZ alone. Representative tracking analyses and heat maps further confirmed these findings, clearly illustrating reduced central zone exploration in both the STZ- and STZ + fingolimod (STZ + FTY)-treated groups (Figure 7). Interestingly, the average speed of movement was highest in the control group when compared to the other experimental groups (SD: 6.527 ± 0.349, STZ: 5.359 ± 0.248, STZ + FTY: 5.260 ± 0.394, F(2,22) = 4.735, *p* = 0.020, η^2^ = 0.301), although this difference did not translate into significant changes in the total distance traveled (Figure 8). A similar pattern was observed regarding grooming behavior: mice treated with STZ groomed themselves less frequently and spent less time on this self-care activity compared to SD controls (climbing (SD: 54.33 ± 3.091, STZ: 35.56 ± 2.042, STZ + FTY: 47.29 ± 2.598, F(2,22) = 13.96, *p* = 0.0001, η^2^ = 0.559); grooming (SD: 5.333 ± 0.727, STZ: 1.889 ± 0.309, STZ + FTY: 2.571 ± 0.751, F(2,22) = 9.434, *p* = 0.001, η^2^ = 0.462)). Administration of FTY partially mitigated this effect (Figure 9). Moreover, a significant reduction in exploratory activity was observed in the STZ-treated animals; however, co-treatment with FTY attenuated this decline, suggesting a modulatory effect of fingolimod on exploratory behavior in this diabetic mouse model.

#### 3.3.2. Elevated Plus Maze (EPM)

**Figure 10:**
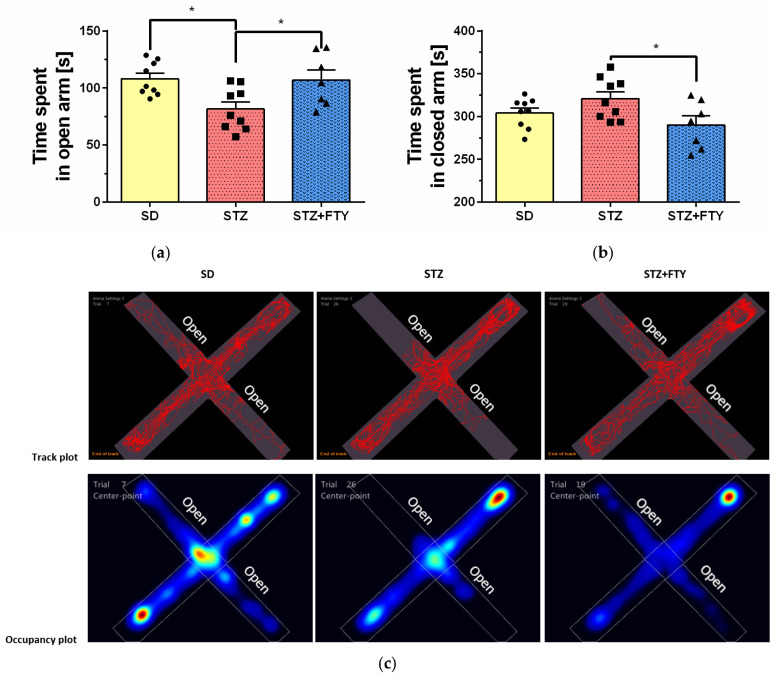
Exploration of the elevated plus maze (EPM) by mice fed a standard diet (SD, *n* = 9), mice on a high-fat diet treated with low-dose streptozotocin (STZ, *n* = 9), and mice additionally treated with FTY720 (STZ + FTY, *n* = 7). Time spent in the open arms (**a**) and closed arms (**b**) of the EPM apparatus, along with representative movement trajectories and occupancy heat maps (**c**). * *p* < 0.05 compared to the respective control groups (one-way ANOVA with Tukey’s post hoc test).

Analysis of the elevated plus maze (EPM) test revealed significant differences between experimental groups in the time spent in the open arms of the maze (SD: 108.0 ± 4.878, STZ: 81.64 ± 6.184, STZ + FTY: 107.1 ± 8.712, F(2,22) = 5.588, *p* = 0.011, η^2^ = 0.337). Mice treated with streptozotocin (STZ) spent significantly less time in the open arms compared to animals from the standard diet (SD) control group and the STZ + fingolimod (STZ + FTY)-treated group (Figure 10). Representative tracking analyses and heat maps illustrating the animals’ location throughout the test corroborated these findings, demonstrating that the STZ group explored the open arms less frequently. This behavior is likely associated with increased anxiety-like symptoms associated with the metabolic and systemic effects induced by STZ administration. Importantly, administration of fingolimod significantly (time spent in closed arm (SD: 304.2 ± 5.815, STZ: 320.8 ± 8.10, STZ + FTY: 290.2 ± 10.52, F(2,22) = 3.494, *p* = 0.048, η^2^ = 0.241)) improved this parameter (times spent in both closed and open arms), suggesting an anxiolytic effect of the compound in the diabetic mouse model.

**Figure 11:**
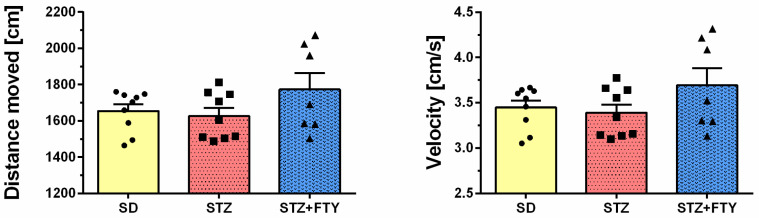
Total distance traveled and movement velocity in the elevated plus maze (EPM) for mice fed a standard diet (SD, *n* = 9), mice subjected to a high-fat diet combined with low-dose streptozotocin treatment (STZ, *n* = 9), and mice additionally administered FTY720 (STZ + FTY, *n* = 7).

No statistically significant differences were observed between groups in terms of the total distance traveled or average velocity during the EPM test (*p* > 0.05), indicating that the observed variations in time spent in the open arms were not due to alterations in locomotor activity but rather reflect changes in exploratory and anxiety-related behavior (Figure 11).

#### 3.3.3. Novel Object Recognition (NOR)

**Figure 12:**
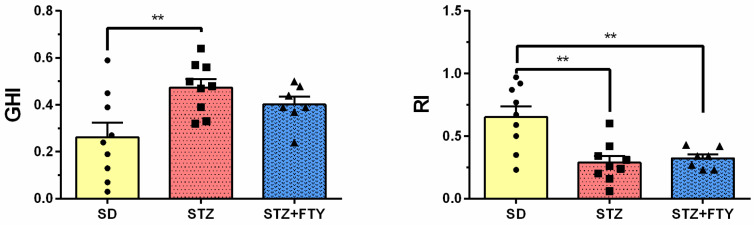
Assessment of parameters, including the global habituation index (GHI) and recognition index (RI), in the novel object recognition (NOR) test for mice fed a standard diet (SD, *n* = 9), mice on a high-fat diet treated with low-dose streptozotocin (STZ, *n* = 9), and mice additionally treated with FTY720 (STZ + FTY, *n* = 7). ** *p* < 0.01 versus respective controls (one-way ANOVA with Tukey’s post hoc test).

We observed that administration of streptozotocin (STZ), both alone and in combination with fingolimod (FTY), altered exploratory behavior compared to animals maintained on a standard diet (SD). Specifically, the recognition index (RI)—defined as the time spent investigating the novel object relative to the total time spent exploring both objects—was significantly higher in the SD control group (SD: 0.652 ± 0.086, STZ: 0.288 ± 0.052, STZ + FTY: 0.323 ± 0.032, F(2,22) = 10.10, *p* = 0.0008, η^2^ = 0.479). In contrast, STZ treatment led to a reduction in this parameter, suggesting impaired recognition memory in these animals. Mice treated with STZ exhibited a significantly lower RI, consistent with deficits in cognitive function (Figure 12). The discrimination index (DI), which reflects the ability to distinguish between familiar and novel objects, did not differ significantly between the STZ and SD groups. In contrast, the global habituation index (GHI) was significantly higher in STZ mice (SD: 0.262 ± 0.062, STZ: 0.473 ± 0.037, STZ + FTY: 0.401 ± 0.033, F(2,22) = 5.477, *p* = 0.012, η^2^ = 0.332), indicating altered habituation in the diabetic group (Figure 12). The elevated GHI in STZ mice indicates impaired habituation, which may reflect diabetes-related cognitive deficits, particularly in memory and adaptive behavioral responses.

Furthermore, no significant differences were observed in the time spent exploring the familiar versus the novel object during the choice phase, nor in the overall time spent examining the objects throughout the entire test.

## 4. Discussion

Type 2 diabetes mellitus (T2DM) is one of the fastest-growing global health challenges, characterized not only by metabolic disturbances but also by a wide range of complications affecting the central nervous system (CNS). Among these, cognitive impairments, memory deficits, and executive dysfunctions are increasingly recognized as significant consequences of chronic hyperglycemia and insulin resistance. Growing evidence suggests that neuroinflammation and disrupted insulin signaling in the brain are key contributors to these neurological deficits in T2DM [30]. Multiple models of diabetes have been created and validated to date, and T2DM research models are the most challenging because they must mimic the impact of the disease on multiple interrelated organs and signaling pathways. In animals, genetic spontaneous and experimentally induced non-spontaneous models of diabetes are used, and the HFD + STZ model is an example of an experimentally induced model [31]. In a recent study, we used an HFD + STZ model that involves the use of a diet rich in fat and low doses of streptozotocin to mimic the pathology of T2DM by induction of hyperglycemia and insulin resistance and/or glucose intolerance. This model replicates the complex and multifactorial nature of human T2DM much better than genetic (spontaneous) models and is not monogenic. Moreover, it combines all the best features from the type 1 diabetes model (using high STZ dose) and the high-fat-diet-induced obesity/pre-diabetes model. Long-term consumption of products rich in fat leads to an excess of free fatty acids (FFAs), which promotes dysregulation of glucose homeostasis and targets insulin-sensitive tissues, including the brain. Fatty acids influence the pathology of metabolic disorders by regulating proteins engaged in sphingolipid metabolism, as well as by being a substrate supply for bioactive sphingolipids [32]. Sphingosine-1-phosphate (S1P) is an interesting bioactive sphingolipid that plays potential roles in the development of diabetes and is a target in the treatment of either diabetes or T2DM-associated conditions [33,34]. SIP, a potential biomarker in many diseases, including diabetes, is synthesized from sphingosine by two sphingosine kinases: SPHK1 and SPHK2 [35]. In the present study, we observed significant upregulation of the *Sphk2* gene in the brain hippocampus that was accompanied by slight upregulation of *Sphk1* gene expression. Although both kinases are responsible for the phosphorylation of sphingosine to S1P, their function and cellular localization differ [36]. Unfortunately, their effect on the brain as well as T2DM progression is still not fully elucidated and may depend on the examined tissue and model of the disease. SPHK2 has been shown to be involved in the pathology of T2DM by promoting insulin resistance and pancreatic β-cell lipotoxicity and dysfunction [37,38].

Fingolimod (FTY720) exerts multifaceted effects on the CNS beyond its well-known immunomodulatory functions. As described by Pournajaf et al. (2022), fingolimod influences neuroprotective and reparative processes by modulating S1P signaling in neurons, oligodendrocytes, microglia, and astrocytes, contributing to reduced neurodegeneration and enhanced remyelination [15]. Although S1PRs are widely expressed in the CNS, S1PR1 has the highest expression level and is involved in the regulation of neurons and synaptic actions [39,40]. S1PRs also appear to play a pivotal role in T2DM pathology. In the present study, we found that the gene expression of *S1pr1* was reduced in both the cortex and hippocampus of T2DM mice, whereas *S1pr3* was upregulated in the hippocampus. The administration of the FTY720 reversed the reduction in *S1pr1* expression, as well as elevated expression levels of *S1pr3*, in the brain cortex of diabetic mice. Our previous study showed that *S1pr1* gene expression is also downregulated in the brain cortex and hippocampus of pre-diabetic obese mice [41]. The current study focuses on measuring mRNA levels in the brain cortex and hippocampus; however, the literature indicates that changes in mRNA and S1P-related signaling protein levels are also observed in both the brain and peripheral tissues. Similarly to our research, Fan et al. revealed that FTY720 can increase the expression of genes encoding S1PR1 and S1PR3 in the retinas of diabetic rats, which were previously downregulated [42]. Certain research suggests that S1PR1 may play a role in regulating food intake, and its levels are reduced in the hypothalamus of HFD-fed rats or mice, as well as in leptin-deficient ob/ob mice [43]. It was recently found that in insulin-resistant rats, despite higher levels of S1P in plasma, the cortical levels of S1P remained comparable to those of the control animals. This effect was accompanied by a lower density of S1PR1 and S1PR4 in nerve-terminal-enriched membranes [44]. S1PR1 and S1PR2 were found to be involved in endothelial vascular dysfunction under high-glucose conditions, where S1PR1 protein and mRNA levels decreased during hyperglycemia, and S1PR2 levels increased [45]. Endothelial cell dysfunction induced by diabetes can lead to neuroinflammation and impairment of the blood–brain barrier, causing cognitive decline and other neurological issues [46].

One of the fundamental metabolic actions of insulin is controlling blood glucose concentration by stimulating glucose transport into specific tissues. Both insulin and insulin-like growth factor (IGF) play important roles in brain functioning and development, and they are responsible for neuronal plasticity and survival, synaptic maintenance, dendritic arborization, myelination, and learning and memory [47,48]. In the hippocampus, IGF-I emerges as a promising restorative molecule for enhancing neurogenesis and improving memory accuracy in aged individuals, and possibly in neurodegenerative pathologies [49]. Disruptions in energy metabolism in the brain, such as insulin/IGF resistance and deficiency, can exacerbate lipid dyshomeostasis by promoting the lipolysis of complex lipids and the production of ceramides. De la Monte et al. (2012) observed a correlation between the aberrant expression of pro-ceramide and ER stress genes and insulin/IGF signaling resistance in the brains of Alzheimer’s disease patients [50]. In the present study, mice treated with streptozotocin (STZ) showed a significant decrease in *Igf1* gene expression in the hippocampus. There is increasing evidence indicating that brain IGF levels are reduced in both types of diabetes [51,52]. Decreased IGF levels may be responsible for cognitive impairment, as synaptic spine density and arborization are reduced in the brains of diabetic rats. Replacement doses of IGF and insulin can prevent loss of total brain protein, widespread cell degeneration, and demyelination. Moreover, IGF-1 can prevent hippocampus-dependent memory impairment [52,53,54]. In the present study, we observed that FTY720 administration reversed the reduction in *Igf1* to the control levels in the hippocampus of diabetic mice. The data about the interactions between IGF-1 and S1PRs is limited, but it is suggested that IGF-1 can stimulate SPHK activity and transactivate S1P1/S1P3 receptors in mouse myoblasts [55].

Glucose is transported into brain cells by specific membrane transport proteins, which include glucose transporters (GLUTs) that belong to the solute carrier (SLC) protein family and are sodium-independent glucose transporters. Moreover, GLUTs’ proper function is crucial for maintaining memory processing. A slight reduction in *Slc2a4* (GLUT-4), which encodes a protein that functions as an insulin-regulated facilitative glucose transporter, was observed in the hippocampus of T2DM mice. GLUT-4 is abundantly expressed in neurons of specific brain regions, such as the hippocampus, and plays an important role in transducing procognitive effects when insulin increases glucose utilization in a GLUT-4-dependent manner [56]. Prolonged systemic GLUT-4 blockade causes insulin resistance and impairs memory acquisition (long-term memory) [57]. As observed in the present study, the elevation of *Slc2a4* and *Igf1* gene expression by FTY720 treatment suggests an important role of S1PR modulation in the regulation of genes encoding proteins responsible for brain insulin action and glucose transport.

Insulin-degrading enzyme (IDE) is a key regulator of glucose levels in the brain, where it is markedly expressed [58]. Mice with a knockout of IDE exhibit age-dependent glucose intolerance, likely due to the hyperinsulinemia-associated onset of insulin resistance [59]. Moreover, IDE is involved in the degradation and inactivation of amylin and glucagon, hormones, growth factors, neurotransmitters, and amyloid beta (Aβ), which implies its engagement in the modulation of diabetes-related cognitive impairment [60,61]. In the present study, we did not observe any changes in the *Ide* expression in both examined structures of T2DM mice, but a significant increase in *Ide* was observed after fingolimod treatment. The observed results may suggest that S1PRs are involved in the regulation of the gene encoding IDE.

In the present study, we observed a significant reduction in sirtuin 1 (*Sirt1*) gene expression in the brain cortex and hippocampus, whereas FTY720 administration restored *Sirt1* gene expression to the control levels in both the cortex and hippocampus of diabetic mice. SIRT1 maintains a variety of cellular processes through deacetylation of histones or transcription factors (such as FOXO, NF-κB, and p53). Moreover, SIRT1 has been proven to be beneficial in diabetes, metabolic disorders, and many age-associated pathophysiological changes, as it is engaged in regulating fat and glucose metabolism, inflammatory response, cell survival, oxidative stress, and adipogenesis [62,63]. Knockout of pancreas-specific SIRT1 in mice impairs islet development, insulin secretion, and glucose metabolism [64]. Conversely, administration of recombinant SIRT1 to the T2DM (db/db) mice prevented body weight gain, islet structure and function, glycemia, and glucose tolerance and restored insulin sensitivity. Additionally, the authors found that SIRT1 can decrease the accumulation of ceramides by inhibiting de novo sphingolipid synthesis in the islets of T2DM mice [65]. Downregulation of SIRT1 in the diabetic brain corresponds with our previous study, where its expression was reduced in obese pre-diabetic mice, as well as with other works [41,66,67,68]. Another important function of SIRT1 is its regulatory effect on inflammatory processes through the deacetylation and inactivation of the p65 subunit of NF-κB, as well as a decrease in proinflammatory cytokines, such as IL-6 and TNF-α [69,70]. Xin et al. (2025) have shown that in cerebral ischemia exacerbated by hyperglycemia, reduced levels of SIRT1/SIRT3 are responsible for an increase in ROS activity and an increase in the inflammatory cascade (presented by elevation of IL-1β, IL-6, and TNF-α) [66]. In the present work, we presented for the first time that modulation (by FTY720) of bioactive sphingolipid signaling can affect the SIRT1 expression in diabetic mice. However, the mechanism responsible for this change still needs to be explained. Gao et al. revealed the SPHK1/S1P/SIRT1 axis, where SPHK1/S1P upregulates SIRT1 and exerts multiple cellular functions to regulate the proliferation and migration of endothelial cells, whereas Ping et al. suggest that S1PR1 may be an important regulator of AMPK/SIRT1 signaling [71,72]. Numerous studies highlight the crucial role of the brain SIRT1 signaling pathway in the development of insulin-resistance-related cognitive deficits in animal models of diabetes [67,68].

Both S1P-related signaling and SIRT1 are involved in regulating inflammatory processes, which are evident during the progression of diabetes and its complications, especially in the brain, and have drastic consequences on its proper functioning. Proinflammatory IL-6, TNF-α, and anti-inflammatory IL-10 gene polymorphisms appear to be associated with a higher susceptibility and to be at greater risk of developing T2DM [73,74]. In the present work, we observed a significant upregulation of proinflammatory genes *Il6* and *Tnf*. The results corresponded with our previous study, where upregulation was observed in both examined structures of obese pre-diabetic mice [41]. Observed results may also suggest that even without cytotoxicity in pancreatic cells induced by STZ, inflammation it is still present in the brain, which is an inherent element of the disease. Elevated levels of proinflammatory cytokines (including IL-6 and TNF-α) have been previously observed in the T2DM brain by other authors [75,76,77]. In the present work, we observed that FTY720 administration successfully reduced gene expression of *Il6* and *Tnf* in the diabetic brain cortex and hippocampus. These results correspond with other T2DM studies where FTY720 reduced protein and/or gene expression of proinflammatory cytokines [42,78].

Results of the other researchers showed a strong association between hyperglycemia, hyperinsulinemia, and neuroinflammation, which is the leading cause of cognitive decline in T2DM [75,79]. In diabetes, the levels of proinflammatory cytokines are elevated in the brain, and this situation can lead to neuronal damage. In the context of diabetes, a condition associated with chronic neuroinflammation and CNS complications, the neuroprotective mechanisms of fingolimod may offer therapeutic benefits. Our paper, utilizing a diabetic model, explores fingolimod’s potential to mitigate diabetes-induced CNS damage, suggesting that its S1P-mediated effects could be valuable in reducing neurodegenerative processes linked to diabetic neuropathology.

Preclinical studies have documented that STZ-induced diabetic rodents develop significant behavioral alterations resembling anxiety, depression, and cognitive impairments, which mirror the neuropsychiatric complications observed in diabetic patients [80]. These behavioral alterations are associated with dysregulation of the CaMKIV/CREB/BDNF signaling pathway in key brain regions involved in anxiety regulation, including the prefrontal cortex, amygdala, hippocampus, and hypothalamus. Specifically, diabetic mice exhibit decreased expression of CaMKIV (calcium/calmodulin-dependent protein kinase IV) in the hippocampus and altered CREB expression in the amygdala and hypothalamus, suggesting that disruptions in this neurotrophic signaling cascade contribute to the emergence of anxiety-like behavior in the STZ-induced diabetic model [81]. These behavioral changes have been linked to elevated neuroinflammation and oxidative stress in brain regions such as the hippocampus and amygdala [82]. Our results from the elevated plus maze (EPM) test confirm these observations and demonstrate that STZ administration significantly increases anxiety-like behavior, as evidenced by a reduced time spent in the open arms compared to control animals on a standard diet. This finding aligns with previous reports indicating that diabetes and associated metabolic disturbances can induce heightened anxiety and emotional dysregulation in rodents [41]. Importantly, treatment with fingolimod ameliorated this anxiety-like phenotype, suggesting a neuroprotective or anxiolytic effect of the compound in the diabetic animal model. The lack of differences in locomotor activity between groups confirms that these behavioral changes are not attributable to altered mobility but rather to anxiety-related responses.

Similarly, in the open field (OF) test, a common assay for assessing exploratory behavior and general locomotion, STZ-treated mice spent less time in the central zone, reinforcing the presence of anxiety-like behavior and reduced exploratory drive. This behavioral pattern is consistent with increased thigmotaxis, a common anxiety indicator in rodents. In the OF test, animals treated with STZ typically exhibit a marked decrease in locomotor activity compared to control groups. Specifically, STZ-treated rodents show a significant reduction in average velocity, reflecting decreased spontaneous movement and exploration. This reduction in locomotion is often accompanied by increased anxiety-like behavior, as evidenced by reduced time spent in the central zone of the arena. The observed decline in velocity and exploratory activity is believed to result from the combined effects of hyperglycemia-induced neuropathy, general malaise, and potential CNS alterations caused by diabetic conditions. These behavioral impairments highlight the impact of diabetes on motor and affective functions and underscore the utility of the STZ model for investigating diabetes-associated neurobehavioral deficits. Fingolimod co-treatment partially reversed these effects, further supporting its role in modulating affective and exploratory behaviors. Notably, although the average speed of movement was reduced in diabetic animals, the total distance traveled remained unchanged, suggesting subtle motor impairments without gross locomotor deficits.

The more pronounced anxiolytic-like effects of fingolimod observed in the EPM test, compared to the less consistent outcomes in the OF and NOR tasks, may be attributed to the distinct behavioral domains targeted by each procedure. The EPM is particularly sensitive to anxiety-like behavior related to open and elevated spaces, making it a well-established model for detecting anxiolytic activity. In contrast, the OF test evaluates both locomotor activity and anxiety-related behavior, which may confound interpretation if the treatment also affects general activity levels. Similarly, the NOR test primarily assesses recognition memory, with only indirect implications for anxiety-like behavior. Moreover, fingolimod has been shown to influence not only emotional but also cognitive and motor functions. Such broader effects may obscure anxiolytic outcomes in tests like OF and NOR that involve exploratory or cognitive components. While our findings show clear effects on anxiety-related behaviors, the cognitive outcomes—particularly in the NOR test—were less robust, with significant changes observed only in the recognition index and not in other measures. This suggests that the cognitive effects may be more subtle or limited in scope. Therefore, these results should be interpreted with caution, and further studies are needed to confirm and extend these findings. Taken together, these findings highlight the importance of using multiple complementary behavioral assays to comprehensively assess the neuropsychological effects of candidate compounds such as fingolimod. Further studies are warranted to clarify the underlying mechanisms and behavioral specificity of fingolimod’s anxiolytic-like actions.

Our findings are in line with those reported by di Nuzzo et al. (2015) [83], who demonstrated the antidepressant-like effects of fingolimod in mice. In their study, behavioral tests such as the forced swim test (FST) and tail suspension test (TST) showed that fingolimod significantly reduced immobility time, indicating antidepressant-like effects likely mediated by S1P receptor modulation in the CNS. These behavioral improvements support the concept that fingolimod can positively influence mood-related behaviors alongside its neuroprotective properties. It has long been established that rodents with STZ-induced diabetes exhibit prolonged immobility in behavioral paradigms such as the FST and TST, which are widely recognized indicators of depressive-like states. These behavioral manifestations are closely associated with a range of neurobiological alterations, including reduced hippocampal neurogenesis, dysregulation of the hypothalamic–pituitary–adrenal axis, and disturbances in monoaminergic neurotransmission [84]. Together, these changes contribute to the affective deficits observed in diabetic models, highlighting the complex interplay between metabolic dysfunction and mood-related neuropathology. Our research is further supported by the results of Magalhães et al. (2024), who demonstrated that chronic oral administration of fingolimod in adolescent mice led to widespread alterations in the hippocampal lipid profile [85]. These lipidomic changes may influence synaptic membrane properties, receptor kinetics, and neurotransmitter release, all of which are essential for proper CNS function. Interestingly, while fingolimod did not affect spatial learning or memory in that study, it did modulate anxiety-like behavior, suggesting a behavioral relevance of the observed molecular alterations. These findings align with our observations in the diabetic mouse model, where fingolimod also exerted measurable effects on behavior.

Streptozotocin (STZ)-induced diabetes is known to affect not only locomotor activity but also self-care behaviors such as grooming. In our study, the STZ group exhibited decreased grooming behavior, which has been linked to stress and impaired well-being. This reduction in grooming behavior may also reflect diabetes-associated impairments in motivation and motor function or increased anxiety levels. Simultaneously, the decline in grooming could be linked to neuropathic changes and CNS dysfunction induced by hyperglycemia, further underscoring the broad impact of STZ-induced diabetes on behavioral repertoires. Our observations are further supported by the work of McCready and colleagues (2023), which provides important insights into the distinct and overlapping mechanisms underlying affective symptoms induced by hyperglycemia and chronic stress. Using an STZ-induced model of hyperglycemia and a chronic mild stress paradigm in male mice, the authors demonstrated that hyperglycemia is primarily associated with depressive-like behaviors, including reduced locomotion in the OF test, decreased grooming, increased immobility in the FST, and diminished marble-burying activity. These behavioral changes were accompanied by increased expression of proinflammatory markers, such as *Bdnf* and *Tnf* in the hippocampus, along with elevated *Il1b* levels in the frontal cortex [86]. Our behavioral results are also consistent with those of De Simone et al. (2020) [87], who investigated the effects of fingolimod in the BTBR T + tf/J mouse model of autism spectrum disorder (ASD). They employed detailed behavioral assessments, including the three-chamber social interaction test and ultrasonic vocalization analysis, to evaluate social behaviors. Fingolimod treatment significantly increased the time spent in social interaction zones and improved communication behaviors, indicating enhanced sociability. Furthermore, the study reported that fingolimod modulated both CNS and peripheral immune responses, suggesting its therapeutic potential in regulating neuroimmune pathways underlying ASD-related behavioral deficits.

Cognitive impairments, particularly in spatial learning and memory, have been reported in STZ-diabetic models. Deficits in the Morris water maze and novel object recognition (NOR) tests correspond with decreased expression of brain-derived neurotrophic factor (BDNF), synaptic plasticity markers, and increased apoptotic signaling in hippocampal neurons [88,89]. In our experiment, the NOR test revealed that STZ administration impaired recognition memory, as indicated by a significantly lower recognition index compared to controls. This deficit highlights the impact of diabetes on cognitive domains such as learning and memory, consistent with clinical observations of cognitive decline in diabetic patients. Although other NOR parameters, including the discrimination index and habituation measures, did not differ significantly, the reduction in recognition index underscores the selective vulnerability of recognition memory processes in this model.

Our results align with those of Hait et al. (2014), who demonstrated that the active form of fingolimod (FTY720-P) inhibits histone deacetylases in the brain, thereby enhancing histone acetylation and facilitating fear extinction memory [90]. These findings suggest that fingolimod may exert behavioral effects through epigenetic mechanisms, which could also be relevant in diabetes-related CNS dysfunction. Sood et al. (2023) recently revealed that FTY720 supplementation of T2DM mice for 30 days alleviates cognitive deficit in T2DM mice by promoting microglial M2 polarization via the pSTAT3-jmjd3 axis, and this effect was independent of blood glucose control [78]. These results correlate with our previous study, in which FTY720 improved the memory of pre-diabetic obese mice [41]. In the present study, we used a different treatment regimen compared to Sood et al. (2023) [78] and did not demonstrate significant cognitive improvement following treatment; however, the drug was effective in improving different parameters. Therefore, the effect of fingolimod on cognitive parameters warrants further investigation.

The study by de Souza and coworkers (2019) further supports the notion that anxiety-like behavior and cognitive alterations, particularly related to fear processing, are prominent features in experimental models of diabetes. Using an STZ-induced diabetic model, the authors demonstrated that diabetic animals not only exhibited pronounced anxiety-like behavior in the EPM, but also showed marked deficits in the extinction of aversive memories and a generalized fear response in a fear conditioning paradigm [91]. Taken together, all these results highlight the multifactorial nature of anxiety-like behavior in diabetic models and the importance of neuroprotective interventions that target underlying molecular dysregulations.

## 5. Conclusions

Our findings demonstrate that STZ induces significant molecular and behavioral alterations, including downregulation of *S1pr1*, *Sirt1*, and *Igf1*, along with upregulation of *Sphk2*, *S1pr3*, *Il6*, and *Tnf*. These changes were accompanied by anxiety-like behaviors and cognitive impairments. Notably, fingolimod treatment reversed several of these molecular changes, enhanced the expression of key genes involved in glucose metabolism and insulin signaling, reduced inflammatory markers, and ameliorated behavioral deficits.

Collectively, these findings support the hypothesis that STZ-induced diabetes in mice leads to increased anxiety-like behavior, reduced exploratory activity, and impaired recognition memory. Importantly, fingolimod appears to exert beneficial effects on anxiety and exploratory behavior, potentially through modulation of neuroinflammatory pathways or S1P receptor signaling, which have been implicated in neuroprotection and neuroplasticity. These results suggest that sphingosine-1-phosphate receptor modulation may represent a promising therapeutic strategy for mitigating neurobehavioral complications associated with T2DM.

Future studies should aim to elucidate the precise molecular mechanisms underlying the behavioral improvements observed with fingolimod and assess its long-term impact on cognitive functions in diabetic models. Moreover, exploring different dosing regimens and treatment durations could provide further insight into its therapeutic potential.

## Figures and Tables

**Table 1:** Number of animals used per group for the gene expression study and behavioral studies.

	SD	STZ	STZ + FTY
Gene expression	6	6	5
Behavioral studies	9	9	7

## Data Availability

The original contributions presented in this study are included in the article. Further inquiries can be directed to the corresponding author.

## Abbreviations

The following abbreviations are used in this manuscript:

BDNF	brain-derived neurotrophic factor
CNS	central nervous system
EPM	elevated plus maze test
FFA	free fatty acid
FST	forced swim test
FTY	FTY720, fingolimod
GLUTs	glucose transporters
HFD	high-fat diet
IGF-1	insulin-like growth factor-1
NOR	novel object recognition test
OF	open field test
S1P	sphingosine-1-phosphate
S1PRs	sphingosine-1-phosphate receptors
SD	standard chow diet
Sph	sphingosine
SPHKs	sphingosine kinases
STZ	streptozotocin
T2DM	type 2 diabetes mellitus
TST	tail suspension test
